# Deep exploration of the TCR CDR3β repertoire specific for viral CD4 T-cell epitopes inside the circulating T-cell repertoire

**DOI:** 10.3389/fimmu.2025.1713225

**Published:** 2025-11-26

**Authors:** Gautier Lhomme, Rémi Giraudet, Valéria Porcheddu, Evelyne Correia, Robert Olaso, Stephane Hua, Bernard Maillere

**Affiliations:** 1 Commissariat à l’Energie Atomique et aux Energies Alternatives (CEA), Institut National de Recherche pour l’Agriculture, l’Alimentation et l’Environnement (INRAE), Département Médicaments et Technologies pour la Santé, Service d’Ingénierie Moléculaire pour la Santé (SIMoS), Université de Paris-Saclay, Gif-sur-Yvette, France; 2 CEA, Institut de Biologie François Jacob Centre National de Recherche en Gé nomique Humaine, Evry, France

**Keywords:** TCR, clonotypes, repertoire, viral, epitopes, HLA class II, NGS, CD4 T cells

## Abstract

**Introduction:**

This study provides an in-depth analysis of the diversity of the CD4 TCR CDR3β repertoire specific to influenza A (HA) and Epstein-Barr virus (EBNA) epitopes.

**Methods:**

Epitope-specific CD4 T cells from 13 healthy donors were enriched using a short-term culture step, isolated based on activation markers, and sequenced for their TCR CDR3β region using high-throughput sequencing. The frequency of each clonotype was then identified within the complete circulating CD4 T-cell CDR3β repertoire.

**Results:**

For both epitopes, the clonotype distribution was markedly skewed, with a small number of highly expanded clones comprising approximately 60% of the repertoire, alongside numerous low-frequency clonotypes. VJ gene usage and motif preferences differed between the two peptides, highlighting epitope-specific TCR selectivity. The response was predominantly composed of private T-cell clonotypes. The proportion of public clonotypes can increase among donors sharing HLA class II molecules and reveals in HLA-unrelated donors the level of TCR promiscuity.

**Discussion:**

Overall, our data demonstrate that CD4 T-cell responses to these viral epitopes are polyclonal and highly personalized. The modest overlap of clonotypes between donors, coupled with a long tail of low-frequency clones, suggests that the full diversity of the epitope-specific T-cell repertoire is likely broader than previously estimated.

## Introduction

B and T lymphocytes play a crucial role in combating various diseases, including bacterial and viral infections and cancer (1, 2). They exert their power by selectively recognizing the antigens, thereby preserving the self-components from deleterious effects. This selectivity is made possible by combining different steps of differentiation and selection of the adaptive immune cells. Antigen recognition occurs through a re-arranged receptor, which is encoded by V D J gene segments that might undergo somatic recombination (3). This unique process ensures that the immune system can respond to an almost limitless variety of pathogens. After a phase of selection by self-antigens in the bone marrow or the thymus, naïve immune cells circulate in the blood and the secondary lymphoid organs. Upon encountering their specific antigen, these cells proliferate and differentiate into effector cells that directly combat pathogens, or into memory cells that provide long-lasting immunity (4). This sophisticated system of diversity generation and selective cell selection enables the adaptive immune system to provide targeted and effective protection against a wide range of diseases.

Owing to the crucial role that the size of the T-cell repertoire plays in generating efficient T cells, T-cell diversity has been assessed using various approaches. Distribution of CDR3 length variants was initially used to evaluate the extent of diversity within Vβ subsets (5). Later, TCR gene amplification and sequencing revealed that there are about 10 million different β chains circulating in the blood (6). More recent studies using high-throughput sequencing indicated similar (7, 8) or greater diversity (9). However, a recent study highlighted the limitation of current methods of estimation and proposed that the size of the T-cell repertoire is likely underestimated (10). The composition and evolution of the TCR repertoire were also analyzed to provide insights into the diversity and dynamics of T cells involved in cancer (11, 12) and infectious diseases (13, 14). In addition to studies on the full T-cell repertoire, deep sequencing has also been used to explore the diversity of antigen-specific T cells (15–18). Since these antigen-specific cells are a small minority among circulating cells, distinguishing them from nonspecific cells remains a significant challenge, but is essential for characterizing their T-cell receptors (TCRs). HLA multimer technology allows tracking and sorting of antigen-specific T cells and can be advantageously combined with sequencing (17, 19). However, this approach has been more successful for the investigation of CD8 cells, rather than CD4 cells (20, 21). The peptide binding register for HLA class II molecules, along with the TCR avidity of CD4 T cells and their frequency, is often suboptimal, resulting in the capture of only a subset of the specific CD4 T cells. (20, 21). Alternatively, antigen-specific CD4 T cells can be characterized after *in vitro* expansion and cell sorting or cloning (15, 16, 18, 22). These steps advantageously increase the T-cell detectability but alter the original T-cell repertoire as the expansion process might not equally favor all T-cell clones, leading to an overrepresentation of certain clones. Although no appropriate control can fully correct for this bias, it needs to be overcome by appropriate approaches to evaluate the clonal frequency within the circulating T-cell repertoire.

In this study, we employed a three-step approach to examine in depth the CD4 T-cell repertoire of viral epitopes. Our analysis focused on the CDR3β, as the TCR β locus contains more V, D, and J segments than the α locus, which lacks D segments, leading to a larger combinatorial and junctional diversity. First, CDR3β sequences from circulating CD4 T cells were sequenced. Next, peripheral blood mononuclear cells (PBMCs) were cultured with the viral epitope to enrich for epitope-specific T cells, which were subsequently sorted and sequenced. Finally, the sequences of these specific T cells were compared to the entire T-cell repertoire to determine their original frequency. By applying this methodology to study CD4 T-cell responses to influenza A (HA) and Epstein-Barr virus (EBNA) epitopes, we aimed to shed light on the diversity and specificity of the CD4 T-cell response. Our results indicate that each individual of the 13 different donors exhibited unique signatures, with highly diverse repertoires composed of dominant clonotypes alongside a large number of unexpanded clonotypes.

## Materials and methods

### Peptides

The influenza hemagglutinin (HA) peptide HA306-318 (PRYVKQNTLKLAT) and the Epstein-Barr virus nuclear antigen (EBNA) peptide EBNA515-528 (TSLYNLRRGTALAI) were from ProteoGenix (France).

### Peripheral blood mononuclear cell samples and HLA genotyping

Blood samples were obtained from healthy donors at the Etablissement Francçais du Sang (EFS), Rungis, France, after informed written consent was obtained from the donors and in accordance with EFS guidelines approved by the Comiteí d’eíthique et de deíontologie de l’EFS. PBMCs were isolated from total blood using Ficoll/Lymphoprep separation (GE Healthcare) and cryopreserved in liquid nitrogen. A portion of the PBMCs was used to purify CD4 T cells using magnetic separation with CD4 microbeads and LS columns (Miltenyi Biotec). Ten million CD4 T cells were frozen (circulating CD4 T cells). For each donor, a minimum of five million PBMCs were used to extract DNA with the Machery Nagel NucleoSpin Blood QuickPure kit for HLA typing by NGS (DKMS, Berlin, Germany) (**Supplementary Table S2**).

### T-cell expansion and activation-induced cell marker assay

Thawed PBMCs were seeded at 2x10^6^ cells/mL in a 6-well plate in the presence of the peptide HA or EBNA (10 μg/mL peptide) in RPMI 1640 medium supplemented with 5% human AB serum (Sigma-Aldrich), 60 U/mL IL-2 (R&D), 100 U/mL IL-4 (R&D) and 1 μg/mL of anti-CD28 (clone 15E8, Miltenyi Biotec). The medium was changed every 2–3 days with the same medium supplemented with IL-2 and IL-4 and, at day 8, the medium was changed for the same medium without cytokines. At day 10, cells were washed and incubated with and without 10 μg/mL peptide and 24 hours later cells were stained with the following antibodies: anti-CD4-VioBlue (clone REA623, Miltenyi Biotec), anti-CD134-PE/cyanine 7 (clone ACT35, Biolegend) and anti-CD137-APC (clone 4B4-1, Biolegend). CD4+ CD134+ CD137+ cells were sorted on a FACS ARIA III cytometer (Becton Dickinson, San Jose, CA, USA) and frozen as a dry pellet (expanded CD4 T-cells). No Fc-receptor blocking step was applied before staining, as cultures contained negligible Fc-receptor-expressing cells; non-specific binding was monitored using isotype controls.

### T-cell receptor repertoire library preparation and sequencing

The sequencing library method was adapted from a published efficient bulk multiplex method (23). Briefly, RNA was extracted from pellets of expanded CD4 cells and circulating CD4 cells using the RNeasy PLUS microkit (Qiagen) or minikit (Qiagen) and quantified with a Qubit 4 Fluorometer (Thermo Fisher Scientific) and RNA HS assay kit (Thermo Fisher Scientific). RNA was reverse transcribed using a TCRB chain constant region reverse primer with the Superscript IV First-Strand Synthesis System (Invitrogen). cDNA was column purified with the Oligo Clean Concentrator kit (Zymo Research). 100 ng (expanded CD4 T cells) or 1000 ng (circulating CD4 T-cells) of TCRB chain cDNA was used as a template for PCR with KAPA2G Fast Multiplex Mix (Roche). Forward TCRB primers were used with a single nested TCRB constant region reverse primer at 0.2 µM per primer. Samples underwent 20 cycles for NGS library preparation for PCR1. For PCR2, 2 µL of PCR1 was added to 18 µL of PCR2 master mix (0.25 µM each i5 and i7 sample barcoding primers) and underwent 20 cycles with the use of Herculase II fusion DNA polymerase (Agilent). PCR2 product was then run on agarose gel (2%) and the desired bands were purified using a GeneJet PCR Purification kit (Thermo Fisher Scientific). Purified libraries were quantified with a Qubit 4 Fluorometer (Thermo Fisher Scientific) and Qubit dsDNA HS assay kit (Thermo Fisher Scientific). Paired-end sequencing was performed on an Illumina Novaseq 6000 for the circulating CD4 T cells and Illumina iSeq 100 for the expanded CD4 T cells with 15% of PhiX control v3 (Illumina).

### Bioinformatic analysis

After sequencing quality filtering, the clean data was aligned against the international ImMunoGeneTics library (https://github.com/repseqio/library-imgt/) using the MiXCR v4.2.0 tool (https://github.com/milaboratory/mixcr). The high-quality reads were further assembled into clonotypes, correcting for PCR and sequencing errors. The downstream analysis was performed with MiXCR, the “immunarch” R package and in-house R script (https://github.com/Gaut1993).

### Statistical analysis

Data and statistical analyses were done in FlowJo 10 and GraphPad Prism 8.3.0. The corresponding figure legends provide the statistical details of the experiments. Spearman correlation analyses were used for comparisons, and Mann-Whitney or Wilcoxon tests were used for comparisons of unpaired or paired data, respectively.

### Graphical illustrations

Certain graphical illustrations were made with BioRender(biorender.com).

## Results

### Setting up a sequencing approach to have in-depth access to T-cell repertoires specific for two viral epitopes

**Figure 1:**
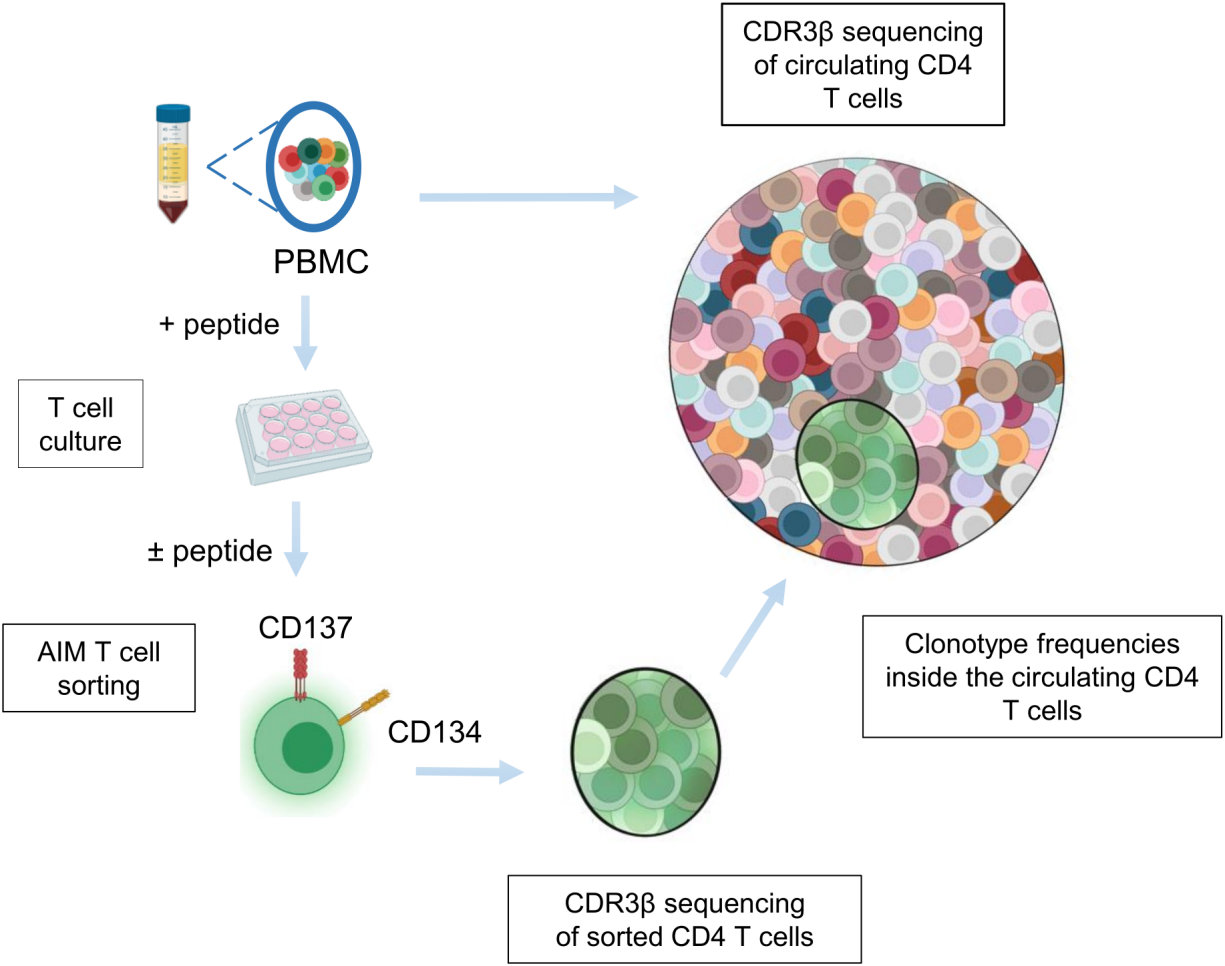
Experimental set-up to capture the antigenic TCR repertoire. Circulating CD4 T cells were purified from PBMCs of each donor. PBMCs were also cultured with peptide HA 306–318 or EBNA 515-528. After 10 days, cultured cells were stimulated with the appropriate peptide and activated T cells were sorted after 24 hours on CD4+/CD134+/CD137+ expression. CDR3β regions of activated T cells (in green) and of circulating CD4 T cells were sequenced. The frequency of the antigen-specific T-cell repertoire was retrieved from the circulating repertoire.

In order to describe in depth the CD4 T-cell repertoire specific to two viral epitopes, we set up an approach combining *in vitro* expansion of antigen-specific CD4 T cells with sequencing of the CDR3β region of their T-cell receptor and the entire T-cell repertoire. This approach comprises three different steps (**Figure 1**). First, CDR3β TCR sequences from circulating CD4 T cells were sequenced. Second, PBMCs of the same donors were cultured for 10 days with each viral epitope to enrich the cell population with epitope-specific T cells. Specific CD4 T cells were then sorted by flow cytometry using the CD134 and CD137 activation markers and their CDR3β TCR region was sequenced. Third, sequences of the specific T-cell repertoire were retrieved from the circulating CD4 T-cell repertoire to obtain their original frequency. We applied this approach to two viral CD4 T-cell epitopes from influenza A (HA306-318 (HA)) and Epstein-Barr (EBNA515-528 (EBNA)) viruses selected from the literature to leverage natural immunity against these common viruses. The HA peptide binds to multiple HLA-DR molecules (24) and participates in the influenza-specific CD4 T-cell response in multiple donors (25) to its HLA-DR promiscuity (24). EBNA515–528 was found to contribute to the T-cell response to Epstein-Barr virus in multiple donors (26). We selected 13 different donors with various HLA typing to gain a broad view of the diversity of the T-cell response to these frequent epitopes. They were retained for the study because they responded to both peptides in IFN-γ ELISPOT, in contrast to other screened donors, who showed no response to at least one of them (not shown).

### The circulating CDR3β TCR repertoire is highly diverse and mainly composed of private clonotypes

**Figure 2:**
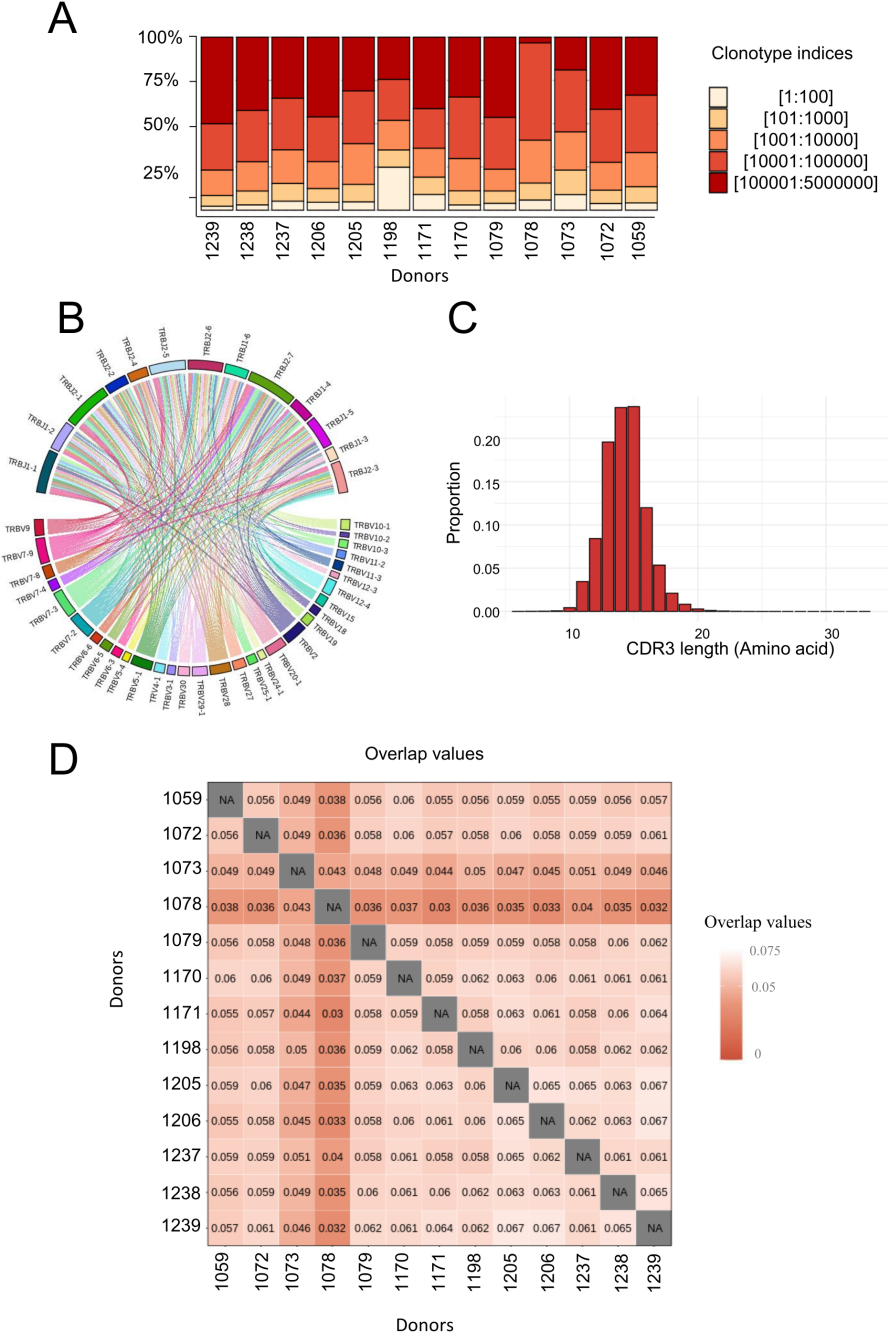
Composition of the circulating CD4 TCRβ repertoire in peripheral blood samples of healthy donors. CDR3β regions of circulating CD4 T cells were sequenced. **(A)** Distribution of CDR3β clonotypes as a function of their relative abundance for each donor. **(B)** Co-occurrence map of TRBV-TRBJ gene usage. **(C)** Amino acid length distribution of the TCR CDR3β. **(D)** Overlap of the 13 healthy donors circulating repertoire (Morisita’s overlap index).

We analyzed the CDR3β T-cell repertoire of the 13 healthy donors by deep sequencing from 10 million circulating CD4 T cells per donor. Overall 4,643,632 circulating CDR3β clonotypes were retrieved after data filtration, varying between 134,960 and 667,858 clonotypes (459,527 ± 160,469) per donor. The top 100 most frequent clonotypes represented between 2.3% and 24.8% of the repertoire for each donor (**Figure 2A**). The top 100,000 clonotypes captured 50% of the total repertoire for 12 of the 13 donors. Almost 50% of the repertoire was composed of very low-frequency clones. A higher number of frequent clonotypes dominated the repertoire of donor #1198 compared to the others. We then explored the VJ gene usage association within the circulating repertoire (**Figure 2B**). We identified a total of 31 V-gene usages and 13 J-gene usages with no statistically significant association. The CDR3β length of the circulating repertoire was also highly diverse, with clonotypes ranging from 6 to 32 amino acids in length, centered around 14–15 amino acids (**Figure 2C**). More than 75% of the circulating clonotypes were between 13 and 16 CDR3β amino acids in length. We also analyzed the frequency of public TCRβ by overlapping every circulating T-cell repertoire between each pair of the 13 donors (**Figure 2C**). Public clonotypes were found in every circulating repertoires, but with minimal overlap of approximately 3 to 6% (**Figure 2D**). It also appears that donors sharing HLA-DRB1 molecules (see **Supplementary Table S1**) do not present a stronger repertoire overlap than other donors.

### Sequencing of peptide-activated T cells reveals a large population of specific T cells, dominated by highly enriched clonotypes

**Figure 3:**
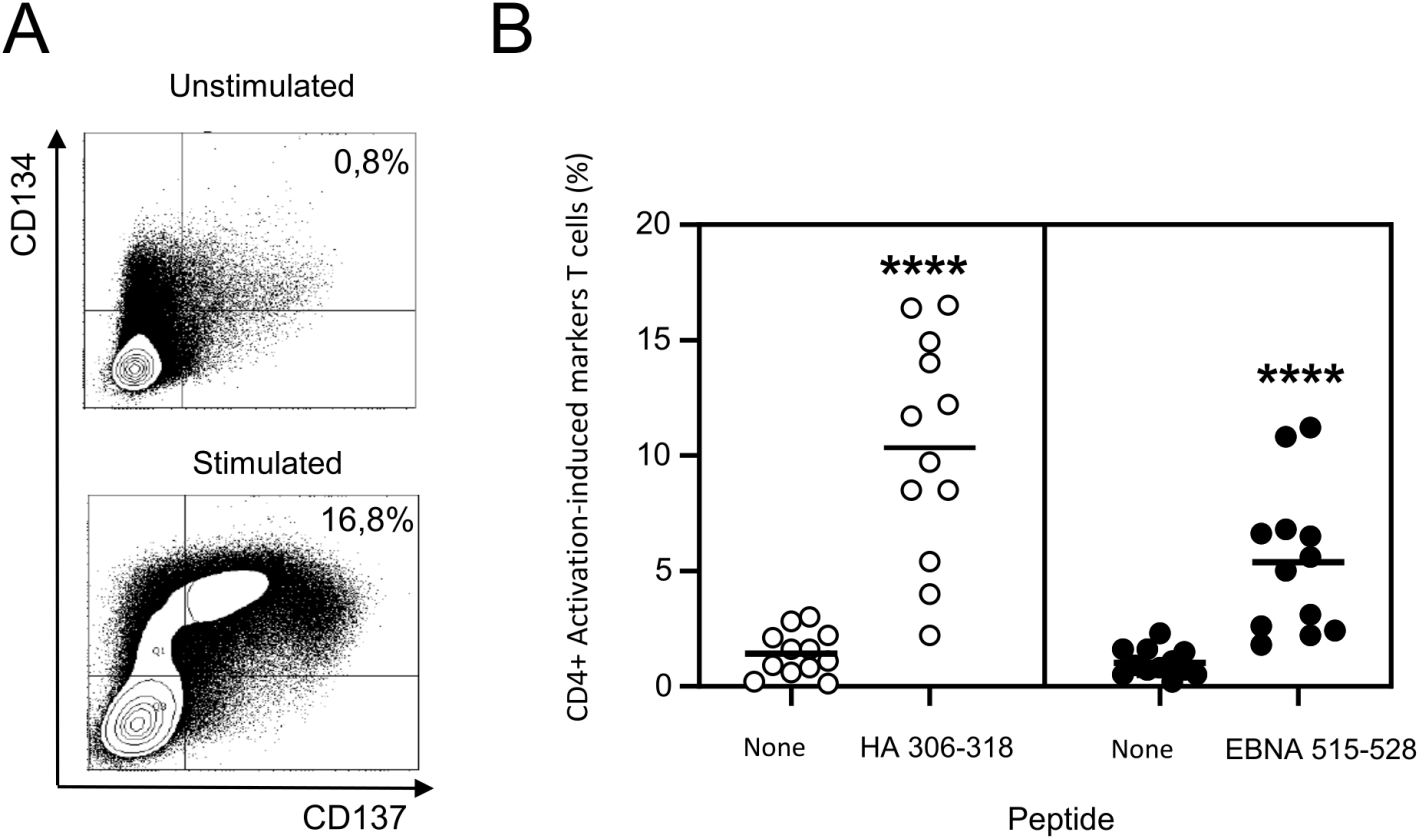
Antigen-specific CD4 T-cell responses of healthy donors. After 10 days of culture of the PBMCs with peptide HA 306–318 or EBNA 515-528, cells were stimulated with the appropriate peptide or non-stimulated and activated T cells were sorted after 24 hours on CD4+/CD134+/CD137+ expression. **(A)** FACS plot examples. **(B)** percentages of activation-induced markers (CD134+ CD137+) CD4+ T cells after stimulation of PBMCs with peptide HA306–318 or EBNA515-528. Statistical comparisons between unstimulated and stimulated groups were performed with the Wilcoxon test (pairwise comparisons). ****p<0.0001.

To enhance the sensitivity of peptide-specific T-cell detection, PBMCs from the 13 donors were cultured with the HA and EBNA peptides for 10 days under culture conditions that were optimized in our previous study (18). This expansion step appeared necessary as CD134 and CD137 expression was inconsistent with freshly isolated PBMCs from two donors showing IFN-γ ELISPOT responses to the HA peptide (not shown). After several washings, cultured cells were stimulated with or without the corresponding peptide and, after 24 hours, expression of activation-induced marker (AIM) (CD4+CD134+CD137+) was assessed by flow cytometry (**Figure 3A**). All donors exhibited AIM T cells upon peptide stimulation (**Figure 3B**). The peptide-stimulated cell population was significantly larger than the unstimulated one for both peptides (p<0.0001), the activation magnitude of the HA-specific CD4 T cells being stronger than that of the EBNA peptide.

**Figure 4:**
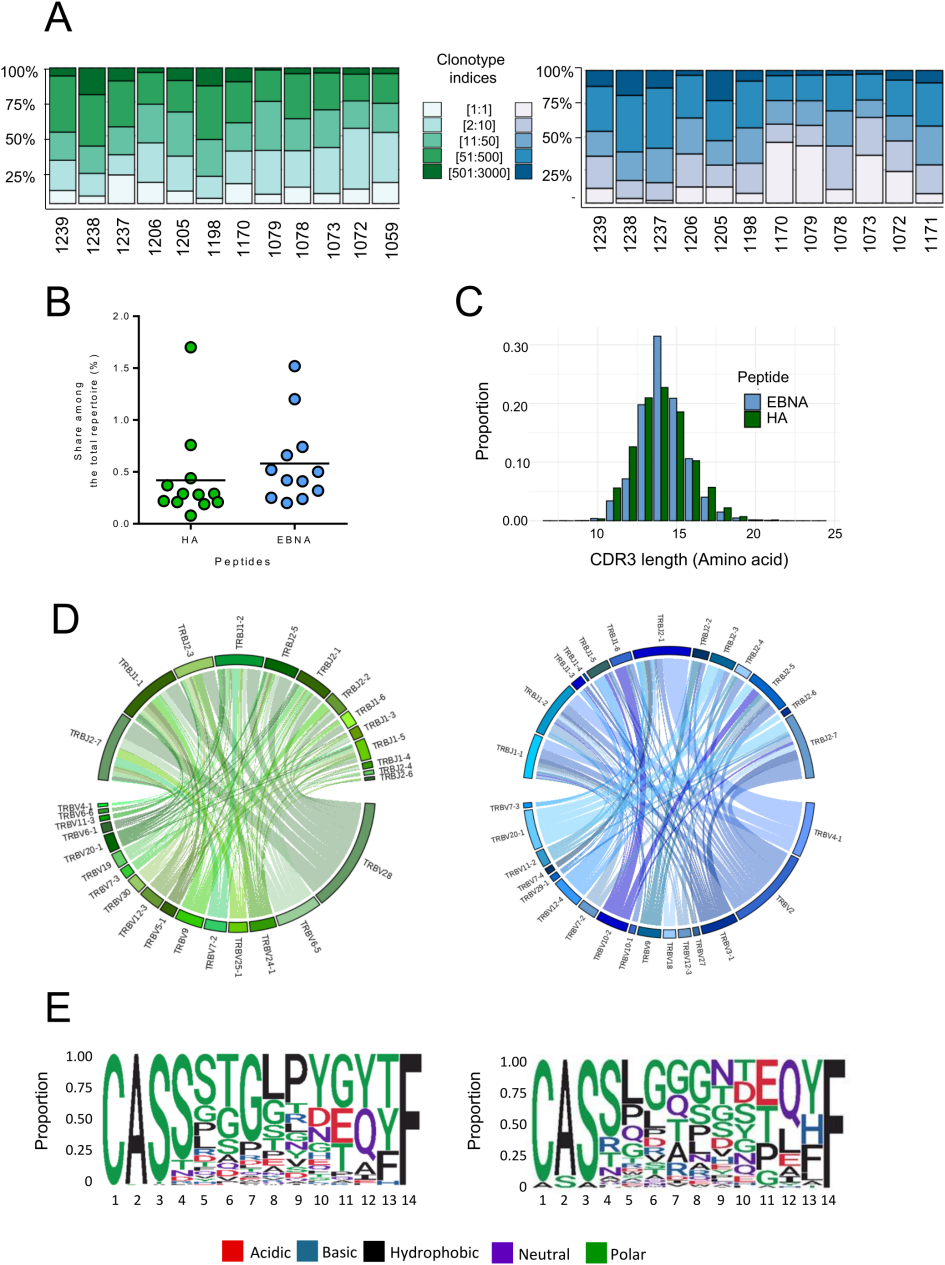
Composition of the epitope-specific CD4 TCRβ repertoires. Analysis of the CDR3β T-cell repertoires was presented for the peptides HA 306-318 (green) and EBNA 515-528 (blue). **(A)** Distribution of CDR3β clonotypes as a function of their relative abundance for each donor. **(B)** Proportion of the epitope-specific repertoire among the total repertoire for each peptide. **(C)** Amino acid length distribution of the antigen-specific TCR CDR3β. **(D)** Co-occurrence maps of TRBV-TRBJ gene usage in both specific antigen repertoires (HA in green and EBNA in blue). **(E)** Sequence logo analysis of the 14-amino-acid CDR3β sequences for the HA and EBNA antigen-specific repertoires.

Afterwards we sequenced the CDR3β TCR repertoire of the sorted CD4+CD134+CD137+ T cells of the 13 healthy donors and evaluated the sequence distribution (**Figure 4**). A total of 18,144 clonotypes were retrieved for the HA repertoire (between 744 and 2911 clonotypes per donor) and 18,291 for the EBNA repertoire (between 1046 and 2971 clonotypes per donor), indicating a similar response size for both peptides. Clonotype proportion showed that the repertoire was dominated by highly expanded clonotypes (**Figure 4A**). A single clonotype dominated each repertoire with a high frequency ranging between 3.9% and 21.2% for HA (mean: 11.2% ± 5.0%) and between 1.9% and 45.8% for EBNA (mean: 17.8% ± 15.4%). The top 50 clonotypes accounted for an average of 63% and 60% of the HA- and EBNA-specific T-cell repertoires, respectively, indicating that the remaining clonotypes, while more numerous, are significantly underrepresented. The sequences were then recovered from the entire T-cell repertoire previously characterized for each donor (see above). The cumulative frequency of specific clonotypes within the total repertoire averaged 0.4% for the HA peptide and 0.6% for the EBNA peptide (**Figure 4B**). This proportion exceeded 1% for one donor with the HA peptide and for two donors with the EBNA peptide. Therefore, the size of the peptide-specific repertoire within the total repertoire appeared significant and comparable for both peptides. A comparative analysis of CDR3β length revealed similarities between the two peptide-specific T-cell repertoires. The length distribution of TCR CDR3β in the peptide-specific repertoire mainly ranged between 13 and 16 amino acids (>10%), 14 amino acids being the most prevalent length (**Figure 4C**). In contrast to CDR3β length, VJ gene usage of the peptide-specific T-cell repertoires showed different preferences (**Figure 4D**). Among all functional human Vβ genes, only the following were detected in the HA-specific repertoire: TRBV4-1, TRBV5-1, TRBV6-1, TRBV6-5, TRBV6-6, TRBV7-2, TRBV7-3, TRBV9, TRBV11-3, TRBV12-3, TRBV19, TRBV20-1, TRBV24-1, TRBV25-1, TRBV28, and TRBV30. Of these, TRBV28 was the most prevalent. Regarding the 13 TRBJ genes, TRBJ2–7 and TRBJ1–1 were the most highly expressed, with the strongest association observed between TRBV28 and TRBJ2–7 in the HA-specific repertoire. In the EBNA-specific repertoire, the following TRBV genes were identified: TRBV2, TRBV3-1, TRBV4-1, TRBV7-2, TRBV7-4, TRBV9, TRBV10-1, TRBV10-2, TRBV12-3, TRBV12-4, TRBV18, TRBV20-1, TRBV27, and TRBV29-1. Among these, TRBV2 and TRBV4–1 were the most prominent. Similar to the HA-specific repertoire, all TRBJ genes were present, with TRBJ1–2 and TRBJ2–7 being the most represented. The major VJ association differed from that of HA and consisted of TRBV4–1 and TRBJ2-1. Both peptide-specific repertoires showed strong VJ preferences, in contrast to the whole T-cell repertoire. Finally, we characterized the clonotype motifs of the two CDR3β T-cell repertoires for the 14-amino-acid CDR3, as it was the most represented in both repertoires (**Figure 4E**). Both motifs exhibited low amino acid diversity per position and shared little similarity, consistent with the strong sequence differences between the two repertoires. The HA motif displayed a higher proportion of hydrophobic amino acids at positions 8 and 9 than the rest of the CDR3β, which contains mainly polar residues. The EBNA motif was characterized by hydrophobic amino acids at position 5 and polar amino acids at position 9. Analysis of CDR3β motifs revealed distinct patterns for each peptide, reflecting strong VJ gene preferences and specific sequence characteristics in each repertoire.

### Peptide-specific repertoires show overlaps between donors but are essentially private

**Figure 5:**
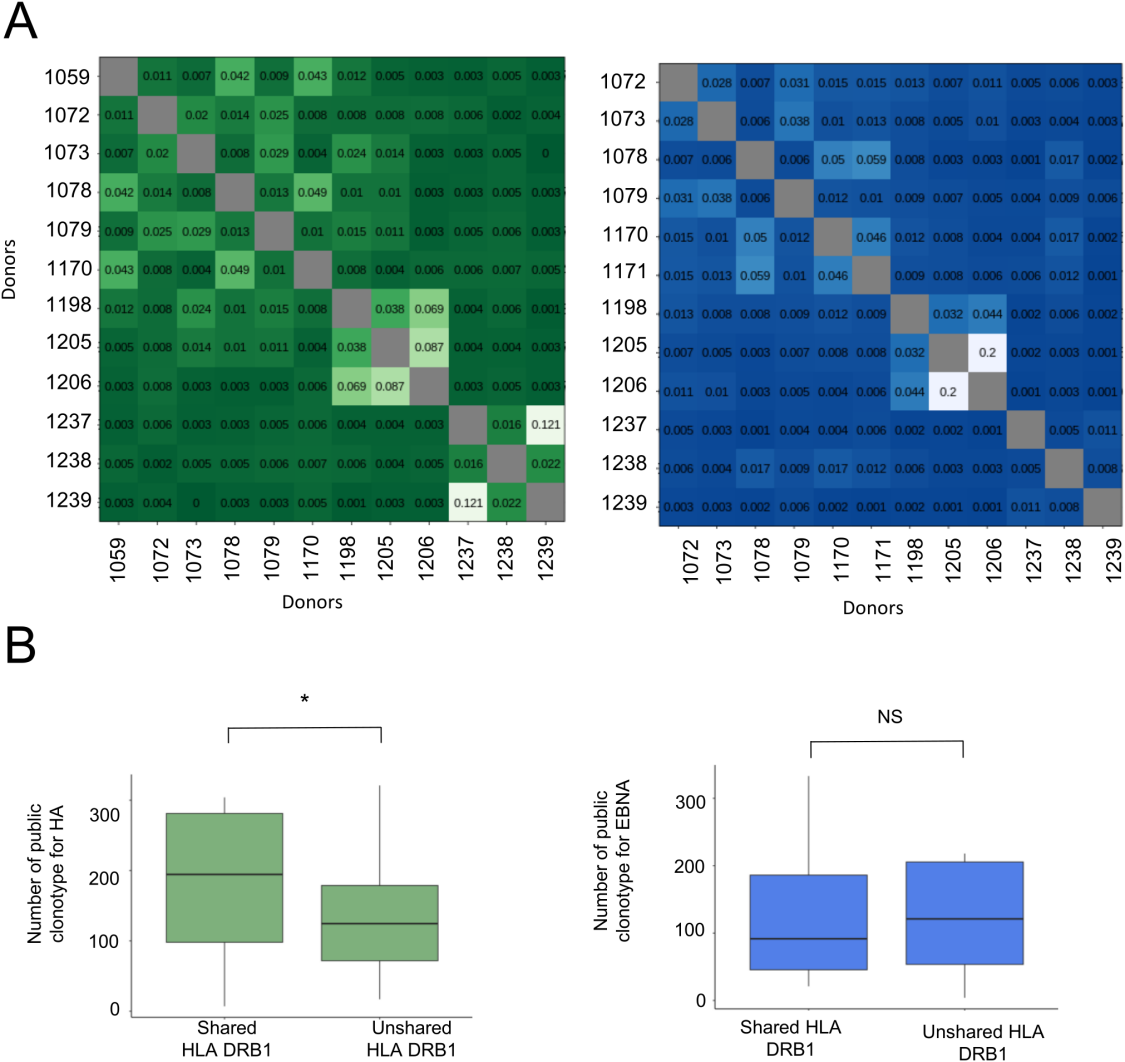
Analysis of the public antigen-specific TCRβ repertoire across the donors. **(A)** Overlap of the antigen-specific repertoire (Morisita’s overlap index) in green for HA and blue for EBNA. **(B)** Number of shared public clonotypes in the antigen-specific TCRβ repertoire with at least one shared or no shared HLA DRB1 allotype.

We also compared the HA- and EBNA-specific CDR3β T-cell repertoires across 13 donors by pairwise overlap analysis (**Figure 5A**). Almost all pairwise comparisons of T-cell repertoires yielded less than 5% overlap and, in many cases, less than 1%, demonstrating that the private repertoire largely dominates for both peptides. Public repertoires were rare. However, donors #1237 and #1239 shared 12% of their HA-specific T-cell repertoire and both possessed the HLA-DRB1*0101 allotype (**Supplementary Table S1**). Donors #1198 and #1206, who also had the HLA-DRB1*0101 allotype, shared 7% of their HA-specific repertoire, but did not share a substantial common HA-specific repertoire with donors #1237 and #1239. None of these donors exhibited significant overlaps across the EBNA-specific repertoires. Notably donors #1205 and #1206, despite having completely different HLA typings, shared 8.7% and 20% of their T-cell repertoires specific for HA and EBNA, respectively. Clonotype sharing is not exclusive to donors with a common HLA-DR molecule, suggesting that some TCRs are promiscuous (27, 28). It was statistically more frequent among donors with at least one shared HLA-DR molecule for the HA repertoire, but not for the EBNA repertoire (**Figure 5B**).

## Discussion

This study provides a comprehensive analysis of the CD4 T-cell response to viral epitopes, employing an innovative approach to explore the diversity of the TCR repertoire of antigen-specific CD4 T cells. This sensitive approach reveals a notably unbalanced, polyclonal and personalized epitope-specific T-cell repertoire.

The methodological set-up allows for a detailed examination of the diversity of the CD4 TCR CDR3β repertoire raised against multiple kinds of antigens. By culturing PBMCs with specific viral epitopes and sorting the cells based on expression of AIMs, this approach selectively enriches for the peptide-specific CD4 T cells, allowing the detection of rare antigen-specific T cells and appears to be highly sensitive. Indeed, we identified between 744 and 2911 HA-specific clonotypes per donor and between 1046 and 2971 EBNA-specific clonotypes per donor, an estimate which is greater than those of previous studies for the HA epitope (29–31). Although PCR amplification and sequencing can lead to multiple sequence errors, the specificity of the sequences is ensured by double sequencing, owing to the low probability that the same error appears in two sequencing runs. This method was applied to a large number of sorted cells and hence provides a detailed view of the diversity compared to single-cell sequencing approaches, which can be used with a limited number of cells (32, 33). Single-cell sequencing of TCR is especially useful to link the epitope specificity to cell phenotype (32, 33) and to preserve the pairing of TCR α and β chains, but it is not appropriate for in-depth investigation of T-cell repertoires. We therefore focused only on the CDR3β sequences, which present a wider combinatorial and junctional diversity than CDR3α.

We first sequenced the CDR3β T-cell repertoire of 10 million circulating CD4 T cells for each donor by dedicating at least 50 million sequencing reads to each sample. This analysis was generally performed with a lower number of cells (7–9), leading necessarily to a lower number of identified sequences than the 134,960 to 667,858 clonotypes we identified per donor. The whole T-cell repertoire appears vast and highly diverse. However, 10 million cells constitute a small sampling to account for the whole theoretical diversity of the 10^12^ circulating immune cells (10). Although the frequencies vary across the different studies, a limited number of clones are much more frequent than the majority of clones, which are unexpanded (7–9, 34). In our study, the top 100 clonotypes represent between 2.3% and 24.8% of the repertoire for each donor, showing considerable variation across the donors. No particular association between V, D, and J segments emerges, while the CDR3β length appears canonical. As already shown (8, 35), the number of common clonotypes across the 13 donors remains low, although we sequenced CDR3β from a large set of 10 million CD4 T cells. This observation suggests that the real diversity might exceed previous estimates (6, 9), as suggested recently (10). In fact, if the total number of clonotypes is limited, by increasing the size of the sampling, the proportion of public clonotypes should increase, which is not the case. Furthermore, we also observed a long tail of individual sequences at very low frequency (clonotype indices from 100,000 to 5,000,000), which together covered up to 50% of the total repertoire. At a scale of an entire human body, they might be present at a substantial number and contribute to immune responses. We therefore suggest that the number of sequenced T cells per individual should be increased to measure with greater accuracy the size of the whole T-cell repertoire.

The main objective of this study was the characterization of the diversity of the epitope-specific CDR3β T-cell repertoire. In contrast to the whole T-cell repertoire, which contains every stage of differentiated T cells, the epitope-specific T-cell repertoire likely corresponded to memory or effector CD4 T cells. It is therefore expected to be more narrowly focused and to show a higher degree of clonotype expansion than the whole repertoire. Indeed, for both the HA and EBNA peptides, the response was characterized by the presence of highly expanded clonotypes. Each repertoire was dominated by a single clonotype, while the top 50 clonotypes covered a mean of approximately 60% of the peptide-specific T-cell repertoires. The reason for this unbalanced distribution is unknown, but it might reflect the predominant expansion of clonotypes with high TCR avidity for the epitope. Nevertheless, a multitude of very low-frequency clonotypes contribute to approximately 40% of the epitope-specific repertoire. Previous studies dedicated to clonality of the specific T-cell repertoire revealed similar curves of distribution, but with a lower number of identified clonotypes, qualifying the T-cell response as oligoclonal (29, 31). Based on our observations, we would prefer to describe it as polyclonal.

We amplified *in vitro* the peptide-specific T cells to characterize them and then calculated clonotype frequencies among the entire T-cell repertoire. We found that the cumulative frequency accounted for approximately 0.5% of the total repertoire for both peptides. This appeared slightly higher than previous estimates (36, 37). Comparison with cell frequency evaluated by tetramer staining (36, 37) should be considered with caution as mRNA content might differ from one cell to another, especially if the cells are differently activated. In the context of non-activated PBMCs, we found that the cumulative frequency accounted for approximately 0.5% of the total repertoire for both peptides. This appeared slightly higher than previous estimates (36, 37). Analysis of VJ gene usage and motif preferences among the T cells responding to both peptides revealed specific patterns consistent with the selectivity of the TCR and the lack of cross-reactivity between the two peptides.

The predominance of private T-cell responses across the donors illustrates the highly personalized nature of immune responses. This has already been documented in multiple studies (38, 39) including in congenic mice (40). Each donor uses a very small part of the large universe of possible recombined TCRs, so it is relatively unlikely that they share the same set of TCRs with another donor. The T-cell privacy therefore confirms the polyclonality of the antigen-specific T-cell response and the difficulty of capturing a large part of the real TCR repertoire with too small blood samples. We acknowledge that our analysis was limited to 13 donors, owing to the experimental design that required deep sequencing of both circulating and antigen-expanded repertoires. Despite this limited cohort size, the observed patterns were consistent across donors, supporting the robustness of our conclusions. Public CDR3β sequences account for a small percentage of the HA- and EBNA-specific T cells according to several studies reporting shared CDR3 sequences (30, 31, 41). Common HLA-DRB1 allotypes were found in our study to increase significantly the overlaps of the TCR repertoire for HA but not for EBNA. Owing to the role of HLA molecules in shaping the T-cell repertoire in the thymus and in driving antigen-specific T-cell activation in the periphery, it would have been expected to be more important. Meanwhile, the substantial occurrence of shared clonotypes among donors without identical HLA-DR molecules hints at the complexity of TCR recognition and its capacity for cross-reactivity (27, 28). It is already known that some TCRs are promiscuous and recognize the same epitope presented by different HLA molecules (27, 28). TCR recognition of peptides presented by HLA molecules has been described as highly selective. TCR promiscuity highlights that HLA sequence variations do not necessarily prevent peptide recognition by T cells, thus revealing different modalities of interaction between HLA complexes and TCR (27). Further, cross-reactivity across completely different peptides (42) or allotypic molecules (43) has also been reported.

Exploration of the HA- and EBNA-specific T-cell repertoire inside the whole T-cell repertoire sheds light on the polyclonal nature of epitope-specific responses, with a small number of highly expanded clonotypes dominating the response and a multitude of low-frequency clonotypes. This wide diversity is also illustrated by the minimal overlap of the T-cell repertoire across the donors. This study also highlights the power of high-throughput sequencing, which allows for in-depth investigation of T-cell repertoire diversity due to its ability to process millions of sequences simultaneously.

## Data Availability

The data presented in this study are available in the GEO repository under accession number GSE309566.
