# Supplementary material for: Deep exploration of the TCR CDR3β repertoire specific for viral CD4 T-cell epitopes inside the circulating T-cell repertoire
Source: Front Immunol. 2025 Nov 26;16:1713225. doi: 10.3389/fimmu.2025.1713225 (PMC12689591; doi:10.3389/fimmu.2025.1713225)
Supplement: Supplementary file 1 [file Table1.docx]

| **Donor ID** | **HLA typing** | |
| --- | --- | --- |
|  | **HLA-DRB1** | **HLA-DRB1** |
| **1059** | 11:01 | 16:01 |
| **1072** | 12:01 | 13:01 |
| **1073** | 03:01 | 15:01 |
| **1079** | 01:03 | 11:01 |
| **1170** | 04:03 | 07:01 |
| **1171** | 08:01 | 13:02 |
| **1205** | 11:01 | 14:54 |
| **1206** | 01:01 | 15:01 |
| **1237** | 01:01 | 03:01 |
| **1238** | 01:01 | 07:01 |
| **1239** | 01:01 | 11:01 |
| **1078** | 04:01 | 11:01 |
| **1198** | 01:01 | 03:01 |

Table S1 : HLA typing of the donors
